# Smoking Enhances Risk for New External Genital Warts in Men

**DOI:** 10.3390/ijerph6031215

**Published:** 2009-03-20

**Authors:** Dorothy J. Wiley, David Elashoff, Emmanuel V. Masongsong, Diane M. Harper, Karen H. Gylys, Michael J. Silverberg, Robert L. Cook, Lisette M. Johnson-Hill

**Affiliations:** 1 Division of Primary Care, School of Nursing, University of California at Los Angeles (UCLA), Los Angeles, California, USA; 2 David Geffen School of Medicine, University of California at Los Angeles (UCLA), Los Angeles, California, USA; 3 Departments of Obstetrics and Gynecology and Community and Family Medicine, Dartmouth Medical School, Hanover, New Hampshire, USA; 4 Division of Research, Kaiser Permanente Northern California, Oakland, CA, USA; 5 College of Public Health and Health Professionals, University of Florida, Gainesville, FL, USA; 6 Department of Epidemiology, Johns Hopkins University, Bloomberg School of Public Health, Baltimore, Maryland, USA

**Keywords:** Smoking/tobacco, Genital warts, Human papillomavirus (HPV), HIV, men who have sex with men (MSM), CD4+ T-lymphocyte count, longitudinal/cohort

## Abstract

Repeat episodes of HPV-related external genital warts reflect recurring or new infections. No study before has been sufficiently powered to delineate how tobacco use, prior history of EGWs and HIV infection affect the risk for new EGWs. Behavioral, laboratory and examination data for 2,835 Multicenter AIDS Cohort Study participants examined at 21,519 semi-annual visits were evaluated. Fourteen percent (391/2835) of men reported or were diagnosed with EGWs at 3% (675/21,519) of study visits. Multivariate analyses showed smoking, prior episodes of EGWs, HIV infection and CD4+ T-lymphocyte count among the infected, each differentially influenced the risk for new EGWs.

## 1. Introduction

External genital warts (EGWs) occur on the penis, scrotum, external anus, perigenital and perianal regions, and are largely due to infection by low-risk *Human papillomaviruses* (HPV) that can be transmitted between sexual partners [1]. Reports are not uniformly supportive in women [2,3], though epidemiologic studies suggest smokers are at higher risk than non-smokers for developing genital warts [4], abnormal cervical cytology [5–7], cervical intraepithelial neoplasia grade 3 or cervical cancer [7–9], and persistent HPV infection [10].

Early observations supported an association between HIV infection, smoking, HPV-related dysplasias, and specific sexual behaviors [11–22]. Several other factors may modify the influence that smoking has upon the natural history of EGWs. Data suggest low CD4+ T-lymphocyte count and higher serum HIV RNA concentrations heighten the risk for both prevalent and incident HPV infections [23]. However, no study before has been sufficiently powered to determine how tobacco use, prior history of external genital warts and *human immunodeficiency virus* (HIV) infection affect the risk for new EGWs, much less how they may interact to cause disease in men.

Exposures that affect EGWs in men likely have bearing on other HPV-related anogenital lesions, e.g., intra-anal infections and anal cancer, especially in the immunocompromised. Some studies report anal warts are associated with a 3- to 5-fold increased risk for anal intraepithelial neoplasia (AIN) in HIV-infected men [24,25] Although EGWs themselves are rarely associated with cancer, their treatment requires significant health-care resources. U.S. insurance data suggest EGWs may cost $150 million annually, that 25 to 34 year old men seek treatment most often and that three visits per episode are required before clearance is achieved [26]. It has been generally accepted that most HPV infections are cleared and that a fraction of cases become persistent. On average, EGWs regress within twelve months of detection [26–28]. Our findings and other studies support that host transcriptional regulation may facilitate viral latency, though the model has not been fully characterized to date [29–33]. Thus, understanding the factors that affect risk for EGWs has importance for basic science and clinical practice, resource planning, and health education efforts.

A latent HPV reservoir has not been identified, and it is unclear how prior EGWs influence the risk for recurrence. Recent data by Strickler *et al*., show that among HIV-infected women, HPV disease recurs in the absence of sexual contact with a partner [23], an indication that recrudescent disease may play a role for some individuals. If we assume that infections are generally self-limiting, controlling for the effect of prior EGWs may introduce bias into our analyses by controlling for the effect of an intermediate variable [34]. Instead, although we hypothesize that infections are recurrent, we cannot discriminate recurrent from new infections in these data. Thus, we estimated the effect of prior episodes of warts to determine whether they influenced the risk for later recurrence or new EGW occurrences. To understand how smoking, HIV infection, CD4+ T-lymphocyte cell counts, and historical EGWs jointly affect the risk for new EGWs, we studied demographic, behavioral, laboratory and physical examination data gathered over as many as nine semi-annual visits from 2,835 gay, bisexual, transgender, and other men who have sex with men (MSM).

## 2. Results

*Descriptive statistics*: About three-quarters of men we studied completed seven to nine MACS study visits (76%, 2,167/2,835). Data were gathered at 7,315 visits where men were HIV-infected and at 14,204 visits where men tested HIV negative. HIV infection is a time dependent covariate; of the infected, 808 men were infected at the first visit (seroprevalent) and 280 became infected between their first and last MACS visit (seroconverters). A total of 1,747 men that tested persistently HIV-negative were included in these analyses.

Smoking, HIV infection characteristics, and a prior history of warts were related in these analyses. Approximately 25% of the study group reported ever smoking across the study period. Additionally, more than half were unaffected, never having shown evidence of EGWs during the first 16 MACS visits *and* reporting no episodes of EGWs during the two years prior to enrollment in the MACS (Table 1). About 60% of HIV-infected men showed at least one study visit where the CD4+ T-lymphocyte count was at least 500 cells/mm^3^ and nearly as many showed no visits where counts fell below 200 cells/mm^3^ (Table 1).

Smokers were 23% more likely than non-smokers to have shown or reported historical incidence of EGWs [risk ratio (RR) = 1.2, (1.2, 1.3)]; also, HIV-infected men more often smoked [RR = 1.2, (1.1, 1.3)] and showed a history of EGWs [RR = 1.9 (1.8, 2.0)] when compared to uninfected men. Additionally, historical evidence of EGWs and CD4+ cell count were associated among HIV infected individuals. For example, men with 200 or fewer CD4+ cells/mm^3^ were more than twice as likely as men with higher CD4+ cell counts to have entered the risk period with a history of EGWs (RR = 2.5, (2.3, 2.9)).

Incident EGWs were reported or diagnosed by MACS examiners for 14% (391/2,835) of men at 3% (675/21,519) of study visits. Nearly half of EGWs were diagnosed by examiners alone (52%, 348/675), 16% (108/675) were reported by participants and confirmed on examination, and 32% (219/675) were reported by participants only. Nearly three-quarters (78%, 357/456) of incident warts were diagnosed on anal/perianal surfaces. On average, the average rate of warts diagnosed or reported annually was 0.07 visits/year (Table 1).

*Multivariate analyses*: Our iterative models showed incident EGWs were associated with smoking, historical EGWs, and HIV infection characteristics, including CD4+ cell count in these data (Table 2). The risk for incident EGWs increased only about 2% with each receptive anal partnership and did not change appreciably for each insertive partnership, i.e., RR = 1.02, (1.00, 1.04) and RR = 1.0, (0.99, 1.01), respectively (Table 2). Further, when we controlled for the potential confounding effects of age, partner number, education, time on study, MACS visit sequence, and repeated observations, the relative risk for EGWs was influenced by smoking, history of EGWs, HIV-infection status and CD4+ cell count (Tables 2 & 3). Our estimates suggest a synergistic relationship between the four latter characteristics. Also, estimates of the risk ratio improved marginally when behavioral and sociodemographic characteristics, repeated measurements, and the order of study visits were incorporated iteratively into the model (Models 1–5, Table 2). For example, when we adjusted for the effect of repeated measurements within subjects and the order of study visits alone in the initial multivariate analysis, men who smoked were 20% more likely to be diagnosed with EGWs than were non-smokers, compared to the 40% inferred in the crude analysis, i.e., crude RR = 1.4 (1.2, 1.7); adjusted RR 1.2 (1.1, 1.5) respectively.

Other characteristics also affected risk for new EGWs. For every year on study, our analyses suggested that the risk for developing new EGWs increased by about 30% (i.e., for each day on study: *B* = 0.0007, SE = 0.0001), and it increased about 2% (0.99, 1.03) for every ten years of age. Risk for new EGWs did not vary by educational status (i.e., compared to men who completed graduate education, [aRR = 1.4 (0.9, 2.2), 1.0 (0.7, 1.4), 1.3 (0.9, 1.7), 1.1 (0.7, 1.7)] for men with ≤12 years, some college education, college graduates, and men with some post-graduate education, respectively; Table 2). Last, we observed a 1% increased risk was associated with each receptive anal intercourse partnership [aRR = 1.02 (0.99, 1.03)] and for insertive anal partnerships, we saw no change in risk [aRR = 1.0, (0.98, 1.02); Table 2].

*Multivariate Analyses with Statistical Interactions*: Our primary interests were to determine how smoking influenced risk for new diagnoses of warts, and whether there was evidence to support recurring or newly occurring infection in these men. Our analyses suggested our risk ratios varied when the statistical interaction between smoking, history of EGWs, and HIV infection characteristics, including CD4+ T-lymphocyte count among the infected, were considered together (Table 3). Specifically, among HIV-uninfected men with no prior history of warts, we found smokers were no more likely than non-smokers to develop new EGWs. However, in 8 of 12 statistical contrasts, there was significantly greater risk for smokers than non-smokers, even after we controlled for the effect of other covariates (Table 3, Models 1–5, comparison of rows 1 vs. 2, and 3 vs. 4). Smoking was most often a significant predictor where men showed a history of prior EGWs; however, for men without a history of warts, these relations were most consistent for HIV-infected men with low CD4+ cell counts (Table 3, footnote comparisons “b”). In these comparisons, we may lack sufficient power to detect a statistically significant difference between risk ratios after we corrected our p-values for multiple contrasts, e.g., for HIV+ men with 200–349 CD4+ cell count, comparing RR = 3.1 vs. 1.9, *p* = 0.01 (Table 3, row 1–2, column 5). Strikingly, risk for new EGWs was greater for men with a prior history of warts. This relationship held irrespective of HIV infection status and CD4+ cell count; e.g., comparing HIV-uninfected smokers with and without a history of EGWs, RR = 2.4 vs. 1.0 (Table 3, rows 2 and 4, columns 1, contrasts “c”).

*Sensitivity Analysis*: Last, we performed a post-hoc analysis where men with self-report for EGWs that were not confirmed by examination were reclassified as unaffected to test whether our inferences might change. This analysis method showed point estimates for the risk ratios of main effects were nearly equal those shown in Model 5, Table 3. Although the confidence intervals for main effects were narrower when self-reports alone were classified as unaffected, the analyses lacked the power to detect statistical interactions between two or more variables.

## 3. Discussion

Although the literature is replete with findings that tobacco smoking is deleterious to human health, few observational studies have been statistically powered to explore these relations as they pertain to HPV-related outcomes in men, and none have examined how smoking interacts with other biological characteristics previously reported as important cofactors for HPV-related outcomes. Specifically, these analyses suggest that smoking, historical occurrences of EGWs, and HIV infection characteristics together differentially influence the risk for new occurrences of genital warts. Further, our analyses show that these relationships are complex and that distilling them into easily understood patient- and provider-directives is challenging.

Other investigators have reported positive associations between smoking, EGWs [35], and other HPV-related atypias. Also, others have found positive associations between smoking and persistence and prevalence of HPV infection in women, suggesting that our observations in men are supportive and consistent with prior findings [10,36]. Daily cigarette smoking and prior history of HPV infection are jointly associated with low-grade cervical dysplasias in women [5]. Further, women diagnosed with cervical cancer and high-grade neoplasias are more likely to smoke than are unaffected women [8,37–39]. However, some data suggest the smoking status has little influence upon risk for high-grade dysplasia when HPV *types* are simultaneously controlled for in the analyses [36,40]. Among men, smoking, low CD4+ cell count, anal HPV-infection and related dysplasias have been positively associated [41,42]. Massad *et al*., recently reported that both current and past female smokers were about 40% more likely to manifest genital warts, after controlling for the effect of HAART; and HIV and HPV infection, and social and behavioral characteristics [43].

The causal pathway that lies between smoking, HPV infection, and its associated cancers is likely multidimensional and complex. It is important to note that the HPVs that are associated with EGWs are rarely associated with malignancy. Nonetheless, smoking may affect EGW development through one or more metabolic pathways that are pertinent to oncogenic HPV infections: the formation of chemically stable DNA adducts [44], oxidation or reduction of potent tobacco-associated carcinogens in epithelial cells [45], modulation of inflammation-associated metaplasia or apoptotic pathways [46,47], by perturbing local immunity [48] and possibly, humoral responses early in HPV infection [49]. Also, data suggest that carcinogens inhaled through tobacco smoke can be detected in downstream genital organ sites [45,50,51].

Nonetheless, some might argue that smoking is generally representative of social risk-taking behaviors that may differentially influencing the prevalence of HPV within sexual networks. Some investigators have reported associations between tobacco use and high-risk behaviors, including higher alcohol consumption and use of illicit drugs [52], unprotected receptive anal intercourse [53,54] and earlier onset of sexual debut [55]. Additionally, some cross-sectional data report smoking prevalence among MSMs to be double that of the general North American male population [56,57]; however, our estimates were more closely approximated to the U.S. mean, 21% [58]. Thus, despite the known biochemical and immune modulatory effects of tobacco, this study cannot rule out the contribution of smoking-related behavioral factors that affect HPV disease.

Whether new EGWs found in MACS men were due to new or recrudescent HPV infections cannot be discerned from these data. The strong role that a history of EGWs plays in predicting new episodes of warts suggests recrudescence may explain some cases. In women, recurrent HPV infections among sexually inactive HIV-infected women suggest recrudescence occurs with some frequency in this population [23]. Nonetheless, recrudescence, infection with new HPV types, and persistence that periodically resulted in clinical warts in these data may each explain these findings.

Epidemiological associations between HPV-related atypias and HIV infection have been reported consistently since early in the domestic epidemic [11–18]. Our analyses suggest that HIV infection and immunodeficiency generally increase the risk for EGWs, which is consistent with the findings of others and our previously reported analyses [19,42,59,60]. Palefsky *et al*., observed that, while HIV-infected men were twice as likely as uninfected men to show progression of HPV-related low-grade to high-grade anal dysplasias, men with fewer than 200 CD4+ cells/mm^3^ were three times more likely to show progression than men with higher CD4+ cell counts [59]. Our data support these observations and suggest that these relations are further modified by the influence that historical EGWs and smoking have upon incident warts. For example, we found that most men with fewer than 200 CD4+ cells/mm^3^ were two and one-half to three times more likely to develop new warts than HIV uninfected, non-smoking men, while the risk was nine-fold greater for smokers with historical EGWs. Although our findings are limited to EGWs, these relations may have bearing on other HPV-related outcomes.

Also, our findings suggest that recrudescence may play a role in EGWs, and by extension, other HPV-related outcomes over time. For example, Strickler *et al*., recently reported that 22% of HIV-infected women reporting no sex partnerships within at least six months of the index visit tested positive for HPV DNA using PCR [23]. Our analyses further support this hypothesis; when we performed statistical contrasts of the risk ratios across HIV and CD4+ cell count strata, men with a prior history of EGWs were more likely to be diagnosed with new EGWs than were men without a prior history of warts.

Our investigation focuses on clinical outcomes and these data cannot speak to the underlying mechanisms of disease, including HPV infection characteristics. Nonetheless, experts generally agree that EGWs can be reliably identified by a trained examiner using bright light and magnification, and clinical trials show a high correlation between this diagnosis and underlying infection with non-oncogenic HPV types (as reviewed by Beutner and Wiley [61]). Clearance of symptomatic warts is thought to be due to cellular immune responses. HIV infection selectively and progressively deletes activated antigen-specific memory T-lymphocytes that modulate these cellular immune responses. This may directly affect an individual’s ability to clear symptomatic warts, specifically, and genital HPV infections, more generally [62–70]. Other data suggest HIV infection shifts cytokine secretion patterns that may influence HPV as well; for example, HIV induces IL-6, a proinflammatory cytokine, that in turn, up-regulates transcription of HPV structural genes [71–75]. IL-6 is found in high concentrations in cervical cancer tissues and has been shown to up-regulate vascular endothelial growth factor (VEGF), and thus vascularization of neoplasias, in a dose-dependent manner [73]. Some HIV structural proteins also appear to up-regulate HPV RNA transcription [72]. Thus, these biological pathways are mechanistically supportive of the reported observations herein.

We found a positive association between historical EGWs and new episodes of external genital warts. These results are consistent with our previously reported relationships between historical occurrences of EGWs and new occurrences of external anal warts diagnosed by trained examiners [19]. Persistent anogenital HPV infection among HIV-infected men and women is well documented [42,60,76,77]. In these analyses, we observed a two-fold increase of risk associated with historical evidence of prior EGWs, even among non-smoking, HIV uninfected men. This suggests, at the very least, that the immunological characteristics that made these men vulnerable to symptomatic warts may persist for some period of time. Whether the EGWs detected among these men represent new infections or recurrences matters little within the context of health care dollars expended for treatment [26]. Currently, most EGW therapies ablate the manifestations of HPV infection and do not directly treat the infection itself [78]. Though oncogenic HPV types were not specifically studied, EGWs and their associated low-risk viruses may be proxy indicators of infection by cancer-causing HPV *types*. The implications of this study have clinical relevance in assessing risk factors for HPV pathogenesis, with the goal of directly targeting screening strategies for those most likely to be affected by HPV-related disease.

There are some limitations to these analyses. The MACS participants are a select group of men and findings based on their data may not be easily generalized to all men or to all MSM, specifically. Approximately one-third of the incident EGWs reported were not confirmed by a MACS examiner. However, prior analyses suggest that, on average, affected men are unlikely to be aware of their EGWs [79]. Thus, these reported occurrences may reflect diagnoses made between MACS visits by community clinicians. Including self-report for EGWs in addition to those diagnosed by a MACS examiner may have introduced information bias, such as recall or interview biases. However, using the more conservative classification scheme to group men with self-reported EGWs together with unaffected men in our post-hoc sensitivity analysis was less informative. Self-report data often cause misclassification, resulting in biases that are difficult to predict [80]. Also, EGWs are an observable clinical outcome resulting from HPV infection, and do not directly confirm the presence or absence of *type-*specific HPV DNA. The MACS has not tested for HPV nor have they gathered specimens that can be tested retrospectively. Thus, we cannot determine whether men with prior EGWs are more often exposed to HPVs, are persistently infected with particular HPV types, or are more likely to be symptomatic given HPV infection. Last, our inability to detect statistically significant differences between some contrasted groups may be related to sparsely populated cells when data were fully stratified. For example, data for 96 visits were available for HIV-infected smokers with ≥850 CD4+ cells/mm^3^ who showed a history of EGWs, and data from 140 visits were analyzed for men who showed no history of EGWs but were otherwise similar. It is likely that we may lack the power to detect a difference between the adjusted risk ratios after we adjusted for the effect of age, partner number, education, time on study, MACS visit sequence, and repeated observations, even if such a difference exists.

These analyses are important because, unlike many prior studies, we have sufficient power to detect complex relationships between HPV infection and important clinical exposures: smoking, HIV infection and historical EGWs. Our findings are consistent with and extend the findings of other investigators that smoking increases the risk for EGWs caused by non-oncogenic HPV infection, and our data suggest but cannot confirm that these factors may similarly affect lesions attributable to oncogenic genital HPV infections. Additionally, HIV and the consequent decline of immunity enhance this risk further as do historical EGWs. Most important, these relations persisted even after we controlled for the effect of the number of insertive and receptive sexual partners each man reported, age, education, study site, and time on study. We should continue to counsel patients to stop smoking. Although we found that each receptive anal intercourse partnership increased risk for new EGWs by a modest 2%, counseling should nonetheless include a recommendation to decrease the lifetime number of sexual partners to avoid exposure to these and other sexually transmitted pathogens, including HIV. Additionally, these data suggest HIV-infected men should be counseled about their enhanced risk for EGWs and other possible HPV-related clinical manifestations that are exacerbated further by tobacco smoking. Future studies should explore these relationships with regard to intra-anal infection and anal intraepithelial neoplasias, and determine whether pharmacological immune reconstitution decreases risk for EGWs and other HPV-related diseases within the context of HIV infection.

## 4. Materials and Methods

*Setting and study group*: The Multicenter AIDS Cohort Study (MACS) is a natural history of disease study that has been described extensively elsewhere [81,82]. Briefly, a total of 5,622 asymptomatic men thought at-risk for AIDS were recruited at four geographic locations between March 1984 and April 1985 [81,82]. At the first MACS visit, men were 37.7 (±7.7) years of age on average; most had completed four years of college, and most reported Caucasian race/ethnicity [81,82]. In 1985, 1,808 men were identified as infected with HIV at their first MACS visit (prevalent positive) when an antibody test became available, and another 559 men have tested positive for HIV (seroconverters) since enrollment.

Self- and interviewer-guided questionnaires were administered semi-annually, approximately 6 months apart, to collect demographic, treatment, medication and behavioral characteristics. Self-report for EGWs has been collected consistently throughout the first sixteen MACS visits. Additionally, trained MACS examiners performed physical examinations at each visit using bright light and magnification where needed. Warts at the anal verge and on perianal tissues were recorded separately from warts on the penis, scrotum and other surrounding genital surfaces. Laboratory data collected at each visit routinely include white blood cell counts and measurements of lymphocyte subsets, e.g., CD4+ cell counts.

Although a consistent core of variables has been collected throughout, the number of different types of sexual partners and specific behaviors were not linked to individual relationships through visit seven. Sexual behavior variables were modified beginning at visit eight to better describe behaviors for individual relationships. Nonetheless, the number of sex acts for specific partners could not be estimated from these data; only the number of partnerships in each six month period could be determined. HIV-treatments that may confound our data were infrequently used during the first sixteen MACS visits. Monotherapy, using nucleoside reverse transcriptase inhibitors alone, was first reported by MACS men as treatment for HIV infection after 1987. For the eighth through 16th visits, no men reported simultaneous administration of more than one antiretroviral medication, including protease inhibitors. More important, the MACS cohort was censored administratively after visit 16, and the scope of data collected for some HIV-uninfected men was limited. Most characteristics of interest varied over time, including the study outcome variable, and were updated for these analyses with each study visit (index visit).

*Study sample*: To maximize power and minimize bias, only data from visits eight to 16 were included in these analyses. The study sample was limited to men who reported no EGWs over the prior six months and were not found to have EGWs by a MACS examiner at their first study visit of the risk period. Additionally, eligible subjects must have completed at least four study visits. A total of 3,030 men completed 22,951 study visits; however, data for 195 men who either reported or were diagnosed with EGWs at their first study visit were excluded from the risk set. Thus, our analyses were limited to data collected at 21,519 semi-annual visits from 2,835 MACS men.

*Study endpoint*: Men included in these analyses began the study period free of symptomatic EGWs, reported no episodes of warts during the prior six months, *and* completed four or more study visits during the study period. Two groups of men met the study endpoint: men with examiner-diagnosed warts on the penis, perigenital, perianal tissues and on the anal verge; and men who self-reported one or more occurrences of EGWs during the six months preceding each study visit. Also, to examine the sensitivity of this classification scheme, additional sub-analyses were performed where men were classified as unaffected if they reported one or more occurrences of EGWs during the six months preceding each study visit, but were not found to have warts by an examiner.

*Exposures of interest*: Four characteristics were of primary interest in these analyses: tobacco use, historical evidence of EGWs, HIV serostatus and, for HIV-infected men, the CD4+ cell count. MACS men self-report tobacco consumption at each semi-annual study visit. For these analyses, men were classified dichotomously as tobacco-users or not at each study visit, reflecting an assumption that tobacco exposure has a short-term effect on risk for new episodes of EGWs. Over the risk period, objective laboratory data summarizing HIV infection status and CD4+ cell counts among those infected, and self-report for tobacco smoking were evaluated and updated at each study visit.

*Other Covariates of Interest*: All eligible men began the risk period asymptomatic for EGWs; thus, to characterize the effect of prior warts on new occurrences, we classified men in three ways. First, those who began the risk period without ever having reported or having been diagnosed with EGWs by a MACS examiner were classified as having no historical episodes of EGWs at the first study visit. Second, men who were diagnosed at the first through seventh MACS visits with EGWs, reported having been diagnosed by a clinician with EGWs between study visits, or reported any history of genital warts at the first MACS visit were classified as having historical episodes of EGWs. Last, men who developed warts during the study period reentered the risk set and were subsequently classified as having a prior history of EGWs. Accordingly, both incident EGWs and prior history of EGWs were treated as time-dependent covariates and were updated at every study visit. Several additional characteristics of interest were time on study and number of receptive and insertive anal sex partnerships, which were abstracted from the data. Additionally, age, education and study site were summarized from the first MACS visit data. For these analyses, age for each man was centered around the mean age of the study group and entered as a continuous variable. Additionally, men who reported 12 or fewer years of education, men with some college, men with baccalaureate degrees, and men with some post-graduate education were compared to men who reported achieving post-graduate degrees.

*Analyses*: Tabular and descriptive statistics were used to explore differences between men with and without historical evidence of prior EGWs, smokers and non-smokers, men with and without HIV infection, and among the infected, five levels of CD4+ cell counts. Inter-current and historical occurrences of EGWs were updated with each study visit. CD4+ cell count groupings were updated at each study visit and men with 850 or more cells/mm^3^ were compared to men with 500 to 849, 350 to 499, 200 to 349, and fewer than 200 cells/mm^3^. Our prior analyses suggest sexual behaviors may confound our estimates because they are associated with both the occurrence of new and historical anal warts, smoking, and HIV infection characteristics [19,83]. Also, to control for confounding of sexual behaviors, the number of receptive and insertive anal partners were each treated as a continuous variable and included in our multivariate analyses. Age and time on study (i.e., number of days between each man’s first MACS visit and the index study visit) were included as continuous variables in multivariable models. Men with post-graduate education were compared to men with fewer than 12 years of education, high school graduates, one or more years of college education but no degree, and men who completed four years of college.

A series of logistic regression models were built to examine the crude risk of being diagnosed with EGWs. Subsequently, a series of generalized linear models with repeated binary responses were developed to determine whether diagnosis of EGWs was affected by the covariates of interest. The SAS GENMOD procedure was used for these analyses [84]. Models were fit by a generalized estimating equation; fits of various models were evaluated by the deviance statistic and were rejected or not by using a 0.05 level of significance [84].

A series of five multivariate models were tested to estimate risk ratios and provide a comparison of the influence that some covariates had upon the risk for being diagnosed with EGWs (Table 1). Our final model estimated the relative risk of developing EGWs at any study visit as a function of smoking, historical EGWs, HIV infection status, and, for HIV-infected men, CD4+ cell counts. Multivariate models adjusted for age, the number of receptive and insertive anal intercourse partners reported for the six-months preceding each study visit (partner number), education, and time on study. We constructed an additive model and then assessed the effect of interaction between smoking, history of EGWs, and HIV infection, including CD4+ cell counts for HIV-infected men. The number of partners men reported was also included in our final analysis. Overall, the model fit well. Additionally, the deviance statistic suggested that the purely multiplicative model was superior (deviance statistic for full multiplicative model versus additive model, *X^2^* = 33.5, *p* = 0.01) [84].

## Figures and Tables

**Table 1.** Smoking, HIV- and EGW-related characteristics of 2,835 MultiCenter AIDS Cohort Study Participants.

Characteristic	N (%)
Smoking	
Never smoked	2,087 (74)
Ever Smokers	748 (26)
Number of Visits Where EGWs were Reported or Diagnosed Among Men Who Reported	
Never Having Had EGWs Before the First MACS visit	
0 wart visits	1,805 (64)
1	126 (4)
≥2	77 (<3)
Ever Having Had EGWs Before the First MACS visit	
0 wart visits	639 (23)
1	117 (4)
>2	71 (<3)
HIV Infected Men	
Number of Visits CD4+ T-lymphocytes < 200 cells/mm^3^^a^	
0 visits	668 (61)
1–3	245 (23)
4–9	175 (16)
Number of Visits CD4+ T-lymphocytes > 500 cells/mm^3^^a^	
0 visits	421 (39)
1–3	292 (27)
4–9	375 (34)
Visits Where EGWs Were Reported Or Detected Over the Study Period	
Mean (SD)	0.24 (±0.75 SD)
Median	0
EGW-positive visits per year	
Mean (SD)	0.07/year
Median	0/year

a HIV-infected men only, N = 1,088 (%)

**Table 2.** Comparison of Five Multivariate Analyses of Relationships Between Behavioral and Demographic Characteristics and Incident EGWs for 21,519 Semi-Annual Study Visits, Adjusted for the Effect Of Repeated Measurements. Adjusted Risk Ratios were Derived from Maximum-Likelihood Logistic Regression Using Generalized Linear Models with Repeated Binary Responses (95% Confidence Limits).

Characteristic	Number of Visits	Number of visits EGWs reported or Examiner Diagnosed N (% row)	Risk Ratio (95% Confidence Limits)^1^				
			Model 1^1^	Model 2^1^	Model 3^1^	Model 4^1^	Model 5^1^
Smoking							
Smokers	5,888	232 (4)	1.2 (1.0, 1.4)	1.2 (1.0, 1.4)	1.2 (1.0, 1.4)	1.2 (1.0, 1.4)	1.2(1.0, 1.4)
Non-smokers	15,631	443 (3)	1	1	1	1	1
History of EGWs							
Yes	6,253	320 (5)	2.0 (1.6, 2.5)	1.9 (1.5, 2.5)	2.0 (1.6, 2.5)	2.0 (1.6, 2.5)	2.0 (1.5, 2.5)
No	15,266	355 (2)	1	1	1	1	1
HIV Infection Status							
HIV Negative	14,204	291 (2)	1^2^	1^2^	1^2^	1^2^	1^2^
HIV Infected			2.2 (1.8, 2.8)^3^				
≥ 850 CD4+ cells	729	27 (4)		2.1 (1.3, 3.4)	2.0 (1.3, 3.3)	2.0 (1.3, 3.3)	2.1 (1.3, 3.3)
500–849	2,202	118 (5)		2.4 (1.8, 3.2)	2.3 (1.8, 3.1)	2.3 (1.8, 3.1)	2.2 (1.7, 3.0)
350–499	1,592	70 (4)		2.2 (1.6, 2.9)	2.1 (1.6, 2.9)	2.1 (1.3, 3.5)	2.0 (1.5, 2.8)
200–349	1,414	78 (5)		2.4 (1.7, 3.3)	2.4 (1.7, 3.4)	2.4 (1.8, 3.4)	2.3 (1.7, 3.3)
< 200	1,378	91 (7)		2.8 (2.0, 4.0)	2.7 (1.9, 3.9)	2.7 (1.9, 3.8)	2.6 (1.8, 3.7)
Number RAI Partners during last 6 months^4^					1.02 (1.00, 1.03)	1.02 (1.00, 1.04)	1.02 (1.00, 1.04)
Number IAI Partners during last 6 months^5^					1.00 (0.99, 1.02)	1.00 (0.99, 1.01)	1.00 (0.99, 1.01)
Time on study^6^					1.00 (1.00, 1.00)	1.00 (1.00, 1.00)	1.00 (1.00, 1.00)
Age^7^					0.99 (0.97, 1.00)	0.99 (0.97, 1.01)	0.99 (0.97, 1.01)
Education:							
≤ 12^th^ grade	2090	85 (4)				1.4 (0.9, 2.2)	1.4 (0.9, 2.2)
Some College	5406	169 (3)				1.0 (0.7, 1.5)	1.0 (0.7, 1.4)
Baccalaureate Graduate	5521	189 (3)				1.3 (0.9, 1.8)	1.3 (0.9, 1.7)
Some Post-graduate Education	2886	94 (3)				1.2 (0.8, 1.8)	1.1 (0.7, 1.7)
Post-graduate Degree	5616	138 (2)				1	1
Study Site							
A	5981	139 (2)					0.6 (0.5, 0.9)
B	4454	134 (3)					1.4 (1.0, 2.0)
C	4518	123 (3)					1.2 (0.9, 1.7)
D	6566	279 (4)					1

1 Adjusted for the effect of repeated measurements for 2,835 subjects and order of semi-annual study visits;

2 Referent group in HIV comparisons;

3 Comparison for HIV infection status, not stratified by CD4+ cell count (cells/mm^3^);

4 Number of “receptive anal intercourse partners” was entered into the model as a single, continuous variable;

5 Number of “insertive anal intercourse partners” was entered into the model as a single, continuous variable;

6 Time on study was entered into the model as the number of days since first MACS visit, as a single, continuous variable;

7 Age, in years, was subtracted from the mean age of the population and entered as a single, continuous variable.

**Table 3.** Multivariate Relationship Between Smoking, Prior History of Genital Warts, HIV-Infection Status and CD4+ Cell Count and Risk for New EGWs, Adjusted for the Effect of Behavioral and Demographic Characteristics. Adjusted Risk Ratios were Derived from Maximum-Likelihood Logistic Regression Analysis Using Generalized Linear Models with Repeated Binary Responses (95% Confidence Limits).

	HIV Uninfected	HIV Infected				
		CD4+ Cell Count				
		≥850	500 – 849	350 – 499	200 – 349	< 200
	Risk Ratios (95% Confidence Limits)					
No History of EGWs						
Non-smokers^a^	1	2.1 (1.4, 2.1)	2.5 (1.7, 3.7)	2.1 (1.5, 3.1)^b^	1.9 (1.3, 2.8)	3.2 (2.2, 4.7)^b^
Smokers^a^	1.0 (0.7, 1.5)^c^	2.7 (1.9, 3.9)^c^	2.0 (1.3, 2.9)^c^	3.9 (2.6, 5.7)b,^c^	3.1 (2.1, 4.6)^c^	3.8 (2.6, 5.6)b,^c^
Prior History of EGWs						
Non Smokers^a^	2.3 (1.6, 3.4)^b^	1.4 (1.0, 3.0)	3.9 (2.7, 5.8)^b^	2.7 (1.8, 4.0)^b^	4.4 (3.0, 6.5)^b^	3.9 (2.1, 7.1)^b^
Smokers^a^	2.4 (1.5, 3.9)b,^c^	8.9 (6.0, 13.1)^c^	7.1 (4.8, 10.4)b,^c^	5.1 (3.4, 7.5)b,^c^	7.4 (5.0, 10.9)b,^c^	6.9 (4.7, 10.1)b,^c^

a Multivariate Risk Ratios are adjusted for the effect of smoking, history of EGWs, HIV infection status, and for the infected, CD4+ cell count, number of receptive- and insertive-anal sex partnerships, and time on study reported at each study visit, and for age centered about the mean of the study group, education, study site, repeated measures (within subject variation), and MACS visit sequence;

b For each column, statistical contrasts are of shaded versus unshaded within the two “History of EGW” categories. Comparing risk ratios of smokers versus non-smokers, summarizing statistical contrasts of risk ratios for men with and without history of EGWs, stratified by HIV infection status and, among the infected, CD4+ cell count. Statistically significant associations shown where, correcting α for 17 statistical contrasts, p<0.003 (for each column, statistical contrasts are of shaded versus unshaded within the two “History of EGW” categories);

c For smokers only, statistical contrasts are of unshaded rows between “History of EGW” categories. Comparing risk ratios of groups of men with and without warts, summarizing contrasts of risk ratios for smokers, stratified by HIV infection status and, among the infected, CD4+ cell count. Statistically significant associations shown where, correcting α for 17 statistical contrasts, p<0.003.
